# Bottlenecks of Motion Processing during a Visual Glance: The Leaky Flask Model

**DOI:** 10.1371/journal.pone.0083671

**Published:** 2013-12-31

**Authors:** Haluk Öğmen, Onur Ekiz, Duong Huynh, Harold E. Bedell, Srimant P. Tripathy

**Affiliations:** 1 Department of Electrical and Computer Engineering, University of Houston, Houston, Texas, United States of America; 2 Center for Neuro-Engineering and Cognitive Science, University of Houston, Houston, Texas, United States of America; 3 College of Optometry, University of Houston, Houston, Texas, United States of America; 4 School of Optometry and Vision Science, University of Bradford, Bradford, United Kingdom; Cardiff University, United Kingdom

## Abstract

Where do the bottlenecks for information and attention lie when our visual system processes incoming stimuli? The human visual system encodes the incoming stimulus and transfers its contents into three major memory systems with increasing time scales, viz., sensory (or iconic) memory, visual short-term memory (VSTM), and long-term memory (LTM). It is commonly believed that the major bottleneck of information processing resides in VSTM. In contrast to this view, we show major bottlenecks for motion processing prior to VSTM. In the first experiment, we examined bottlenecks at the stimulus encoding stage through a partial-report technique by delivering the cue immediately at the end of the stimulus presentation. In the second experiment, we varied the cue delay to investigate sensory memory and VSTM. Performance decayed exponentially as a function of cue delay and we used the time-constant of the exponential-decay to demarcate sensory memory from VSTM. We then decomposed performance in terms of quality and quantity measures to analyze bottlenecks along these dimensions. In terms of the *quality* of information, two thirds to three quarters of the motion-processing bottleneck occurs in stimulus encoding rather than memory stages. In terms of the *quantity* of information, the motion-processing bottleneck is distributed, with the stimulus-encoding stage accounting for one third of the bottleneck. The bottleneck for the stimulus-encoding stage is dominated by the selection compared to the filtering function of attention. We also found that the filtering function of attention is operating mainly at the sensory memory stage in a specific manner, i.e., influencing only quantity and sparing quality. These results provide a novel and more complete understanding of information processing and storage bottlenecks for motion processing.

## Introduction

**Figure 1:**
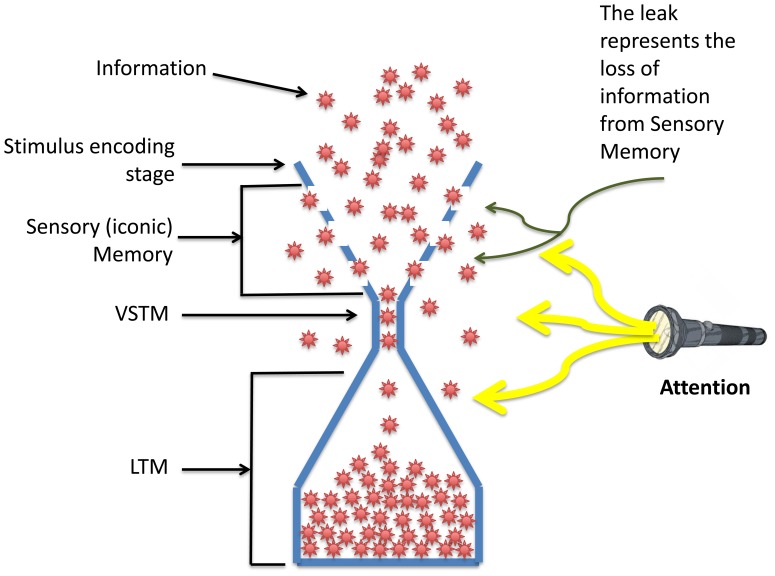
Leaky hourglass analogy for information processing and storage capacity. The initial information processing stages, such as the retina and the early areas of visual cortex, have a parallel structure that allows them to process a large amount of information. The contents of this stage are transferred to sensory storage which has a large capacity but limited time-span of storage. In the leaky hourglass analogy, the limited time-span of storage is depicted by the leak of information from the hourglass. VSTM, which is the visual component of working memory, has limited capacity and represents the major bottleneck of the hourglass. LTM, represented by the bottom half of the hourglass, can accumulate a very large amount of information throughout our lifespan. Finally, the selection and filtering functions of attention can potentially impose their limits upon these three stages.

A fundamental challenge in visual and cognitive sciences is to understand the factors that limit our ability to process and remember the continuous stream of information impinging on our visual system. The traditional conceptualization of capacity limits can be characterized by a “leaky hourglass” analogy, as shown in Figure 1. In the early stages of visual processing, stimuli falling at different retinotopic loci are processed in parallel. Due to this massive parallelism, the early stages of stimulus processing are thought to have a very large capacity. The contents of the information extracted by these stages are stored in sensory (iconic) memory. Iconic memory has been characterized as a high capacity memory whose contents decay within few hundred milliseconds [1]–[3]. The leaky part of the hourglass analogy describes this rapid loss of information. The next level of memory, Visual Short-Term Memory (VSTM), which is part of working memory, has a retention period on the order of seconds. However, VSTM is very limited in capacity [4]–[8]. Finally, the contents of VSTM are transferred to Long-Term Memory (LTM). The retention period of long-term memory can be years or even our entire lifespan. The capacity of LTM is very large since we can accumulate enormous amount of information throughout our lifespan into our LTM.

Given this traditional characterization of stimulus processing, encoding, and memory processes, the prevalent view is that the major information bottleneck resides in VSTM [4], [5], [9], [10]. As a result, most of the recent studies addressing this issue focus exclusively on VSTM, with the debate being centered around the components of working memory [9] and whether a fixed number of discrete slots or a sharable but finite resource imposes the limits at the stage of VSTM [7], [11]–[18]. However, in contrast to the widely held assumption of “VSTM as bottleneck”, single unit recordings from monkey frontal and parietal cortices suggest that the major loss of information occurs *during* rather than after stimulus presentation [19]. A similar conclusion was reached in a recent study, which showed significant information processing limits for stimuli still in view [20]. These findings suggest that a significant information bottleneck may lie at a stage prior to the engagement of VSTM. Other lines of investigation suggest that working-memory systems may include both high-level cortical areas (e.g., prefrontal cortex) and lower-level sensory cortex (e.g., V5/MT) [8], [21]. Given the involvement of lower-level sensory areas, it is possible that significant bottlenecks also occur *during* stimulus encoding prior to stimulus registration in VSTM.

Historically, many studies of iconic memory used easily discernible stimuli so that performance was high when measured during or immediately after stimulus presentation. For example, in his original study, Sperling [3] used arrays of letters and numerals that provided 80% to 90% correct performance (corrected for guessing) when the cue appeared before the stimulus. This paradigm allowed the analysis of capacity limits for iconic memory independently from the capacity limits of mechanisms that process the stimulus while it is in view. Let us note, however, that performance at zero cue delay was not uniformly high in all studies; for example, in Treisman et al.’s study [22] on iconic memory for shape and motion, performance at zero cue delay was in the range 75–78% correct, equivalent to about 50–56% correct when corrected for guessing. Under normal viewing conditions, stimulus encoding and memorization need to work in synergy and, therefore, it is necessary to understand how each stage imposes its limits during their joint operation. Thus, the first goal of our study was to analyze systematically the information processing limits of the visual system from stimulus encoding to stimulus registration in VSTM.

Under normal viewing conditions, a staggering amount of information is presented to our visual system and only a subset of this information is selected for further processing. Attentional mechanisms enhance processing of selected “targets” (*the selection* function of attention) and actively suppress the processing of “distractors” (*the filtering* function of attention) [23]–[33]. As another major constraint on information transfer, attention can potentially impose its limit from the early to late stages of information processing as depicted in Fig. 1. The second goal of our study was to investigate how attention influences the processing of information and its storage in memory.

Perception is an active process that involves eye movements and attention working in tandem. Saccades rapidly reposition the fovea on regions of interest. Information extracted from each fixation (“glance”) is integrated into the complex set of ongoing cognitive processing, such as LTM, goals, expectations, and emotions, to name a few. Given that a glance constitutes a fundamental building block of this process, a final goal of our study was to analyze the bottlenecks that limit the information processing within a single glance.

The specific perceptual feature that we have chosen to study is motion perception. Motion is a fundamental perceptual dimension. From infancy to adulthood, it plays an essential role in vision. The significance of motion is also reflected by the specialization observed in the cortex. In primates, directional selectivity starts as early as V1 and neurons in areas V5/MT and MST exhibit a strong preference for motion (e.g. [34]–[36]). Several studies have shown that motion information is stored in sensory memory and VSTM [14], [22], [37]–[47]. Here, we extend these studies to examine capacity limits for motion, both in terms of the quality and quantity of information.

The current study is relevant also for other types of investigations that use stimuli similar to those used here. For example, sensory memory has a major influence on performance in tasks that involve tracking deviations in trajectories of multiple moving objects, a task referred to as multiple trajectory tracking (MTT) [41], [45], [47]. In these MTT studies, deviations can be detected in as many as 4 or 5 trajectories if the deviations are large, but only a single deviation can be detected reliably if the deviations are small. In the traditional multiple object tracking (MOT) paradigm, subjects track a set of targets over time in the presence of a set of identical distractors [48]. Several differences exist between the task used here and those in traditional MOT studies – the stimuli in MOT are presented for longer durations, usually 5 to 10 seconds, in more complex trajectories and the task of the observer is usually to report the identity (target or distractor), as opposed to the direction of motion, of an object probed at the end of each trial. Here too, as many as 4 or 5 objects are typically tracked concurrently, though as many as seven can be tracked if the objects move very slowly [49]. The results of MOT studies usually have been interpreted in terms of: hypothetical pre-attentive indices or pointers that are attached to tracked objects and move with them (FINSTs in [48]); flexibly allocated resources (FLEXs) or capacity of attention [49]; or visual working memory [50], [51]. The roles of encoding and sensory memory in MOT have not been systematically investigated and to properly interpret MOT performance we need to understand these contributions as well. MOT requires the constant updating of location information for the objects taking into consideration their motion. In addition to location information in the MOT stimulus, the available motion information also aids observers during tracking [52], [53]. If MOT is accomplished by a serial process [45], [47], or one serial process in each hemi-field (Alvarez & Cavanagh [56], [57] reported independent tracking in the two hemi-fields) then it is critical that the motions of tracked objects be buffered in sensory memory until they have been accessed by this (or these two) serial process(es). In contrast, if MOT is accomplished by a multi-focal parallel process (e.g. [56], [57]) with each tracked object being a focus of attention, then, when the motions of tracked objects are sometimes briefly occluded by other objects [52], [58], the buffering of motion information in sensory memory and perhaps short-term memory is vital to the continuation of successful tracking. The current study investigates the nature of memories used to buffer and store motion information when multiple moving objects are presented to the visual system and the temporal dynamics of these memories. These results provide important information for understanding the temporal constraints on the cycle-time for any hypothesized serial process for MOT (see [54], [55]) or for understanding the temporal limits of any hypothesized parallel process for MOT when this process has to deal with occlusions.

## Methods

### Equipment

Stimuli were presented on a 20 inch NANAO FlexScan color monitor with a resolution of 800×600 pixels and were created using a Visual Stimulus Generator (VSG2/3) video card (Cambridge Research Systems). A head and chin rest were fixed at a distance of 1 m from the monitor. The entire size of the display screen was approximately 23×17 deg. Each pixel subtended approximately 1.7 minutes of visual angle. Stimuli were presented at a video frame rate of 100 Hz.

### Observers

Four observers, including one of the authors, participated in the experiments. All observers, with the exception of the author, were naïve to the specific purposes of the experiment. Observers’ ages ranged from 23 to 30 years old (Observer OEK: 23, observer EEK: 30, observer DHL: 28, observer MON: 25). All experiments were conducted according to a protocol approved by the University of Houston Committee for the Protection of Human Subjects and in accordance with the federal regulations, 45 CFR 46, the ethical principles established by the Belmont Report, and the principles expressed in the Declaration of Helsinki. Participants provided their written informed consent following the consent procedure approved by the University of Houston Committee for the Protection of Human Subjects.

### Experiment 1 (Stimulus Encoding Stage)

**Figure 2:**
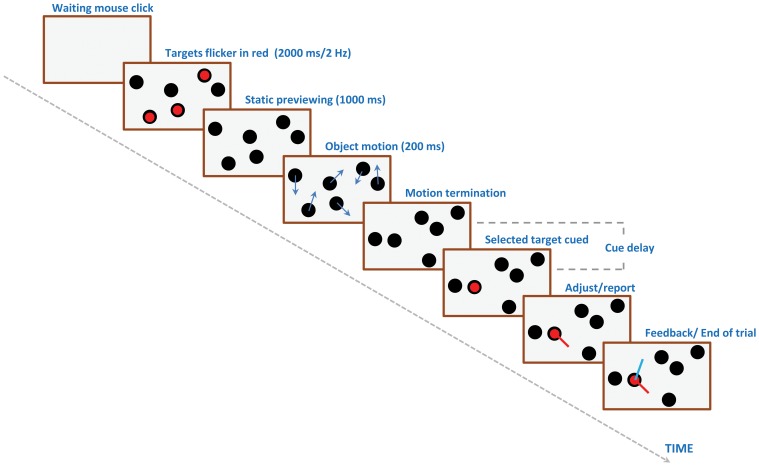
Schematic depiction of the stimulus and sequence of events on each trial. In the first experiment, cue delay was fixed at 0

Figure 2 shows the stimulus display. A trial started with the subject’s mouse click, following which a variable number of objects appeared on the screen. The objects were circular disks with a diameter of 1 deg visual angle and a luminance of 7 cd/m^2^ on a 65 cd/m^2^ background. The initial positions of the objects were chosen randomly, but without spatial overlap. The objects remained stationary for 2.5 s after they appeared. A randomly selected subset of the stationary objects was marked as “target” by flashing (at a frequency of 1 Hz for two seconds) red dots at each object’s center. The remaining unmarked objects were “distractors”. After the stationary period, all objects moved along linear trajectories, each with a randomly chosen direction, such that the angle between the directions of motion of any two objects was greater than 10 degrees. Object speed was 5 deg/s and motion duration was 200 ms for all trials. Objects did not interfere with each other during their linear trajectory movement and their velocities remained unchanged even if they moved across each other. Objects bounced off the edges of the display screen by reversing either the horizontal or vertical component of their velocity. The duration of motion was fixed at 200 ms to minimize the likelihood of eye movements or other cognitive strategies during viewing and thereby to limit the study to the basic information available within a single glance. As mentioned before, the stimuli used in the current study are variations of those used in traditional Multiple Object Tracking (MOT) studies (e.g., [48]). In traditional tracking studies, the duration of motion is typically several seconds and objects undergo complex random motion trajectories. This requires the maintenance of object identities over several seconds during which VSTM and cognitive strategies (such as forming virtual groups [59], shifting gaze, and/or attention towards the center of global motion [60], [61]) play a role. In a previous study with a stimulus paradigm similar to the one used here [43], we tested two stimulus durations, 200 ms and 5 s. Stimulus duration did not have a significant effect for 2 of the 3 observers, while one observer showed better performance at the shorter stimulus duration. This difference may be due to different strategies used by different observers. By keeping stimulus duration short, we sought to minimize the involvement of VSTM and cognitive strategies, as well as eye movements, during stimulus presentation.

*Immediately* after the offset of the motion, one of the targets was cued using a red dot. The observer’s task was to report the direction of motion of the cued target using the computer mouse. In cases where the cued target was one of the objects that had just bounced off the screen edge, the observers were required to report the target’s final direction of motion. When the observer moved the mouse to respond, it caused a direction cursor to appear. This was a line segment extending from the center of the cued target towards the cursor representing the screen-position of the mouse. This line segment was adjusted by the observer to report the direction of motion of the cued target. The mouse controlled the direction indicator with a 1 deg resolution. After the observer’s response, an additional direction indicator appeared to indicate the true direction of motion. The difference between the observer’s reported direction and the true direction of motion was the dependent variable and all statistics were carried out on these error measures. One of the models we used to analyze data contains a “misbinding” term to account for the cases where the subject erroneously reports a non-cued item instead of the cued item. The use of this model necessitated the inclusion of the aforementioned 10 degree lower-bound for the angle between the directions of motion of any two objects.

Let T ( = 1, 3, 5, or 9) and D ( = 0, 3, 5, or 7) denote the number of targets and distractors, respectively. This yielded 16 (4×4) combinations of numbers of targets and distractors. These conditions were blocked so that in each block the number of targets was fixed (e.g. T = 3) and the number of distractors was varied according to D = 0, 3, 5, or 7. The number of distractors was randomized from trial-to-trial so that when, for example, T = 3, a given trial was one of the four possible conditions (T = 3, D = 0), (T = 3, D = 3), (T = 3, D = 5), (T = 3, D = 7). The baseline condition had T = 1 with a variable number of distractors.

All 16 conditions were run. Each condition had 20 trials and was repeated 5 times. Totally, there were 1600 (16×20×5) trials. These trials were run in 4 different blocks, each with a different value of T ( = 1, 3, 5, or 9). Blocks were counterbalanced across observers. Before every block, observers performed 30 trials as a training session.

### Experiment 2 (Memory Stages)

The design was similar to Experiment 1 with the following differences: the cue indicating the target for report appeared with one of seven cue delays (cue delay = 0, 50, 100, 250, 500, 1000, or 3000 ms), and T ( = 1, 5, or 9) and D ( = 0, 5, or 7) were modified slightly. During the delay interval, all objects were identical and stationary, displaying the final frame of motion. The seven cue delay values and three values for the number of distracters were combined randomly for a fixed value of T. For each observer, and for each target condition, this yielded 21 (7×3) conditions, with 100 trials per condition, totaling 2100 (21×100) trials. These trials ran in 3 different blocks, each with a different number of targets (T = 1, 5, or 9). Block order was randomized for each subject. All four subjects from Experiment 1 participated in this experiment.

In an experiment with a limited number of stimulus categories, subjects can code the stimulus verbally and use “the phonological loop” to extend stimulus storage [62], [63]. For example, in a study with only four possible directions of motions, up, down, left, right, subjects can code target directions verbally by using “up”, “down”, “left”, and “right” to rehearse the intended responses in the phonological loop. The use of a very large number of potential directions of motion (360) makes the verbal coding for the phonological loop virtually impossible. To assess empirically the potential effects of verbally encoding and rehearsing the stimuli, we ran a control experiment in which one subject re-run Experiment 1 while repeating continuously the word “the”. Such repetition, termed *“articulatory suppression”* is known to prevent stimulus rehearsal [62], [63]. Results for the blocks with and without phonological repetition were similar (F(1,24) = 0.467, p = 0.501, η_p_^2^ = 0.19).

### Statistical Analyses

Data were analyzed using repeated-measures ANOVA with Huynh-Feldt correction for sphericity, as appropriate. In addition, we provide in Supplementary Information Bayesian analyses derived by using the repeated-measures ANOVA procedure described in Rouder et al. [64].

## Results and Discussion

### Experiment 1: Stimulus Encoding Stage

In the first experiment, a *single* item from the targets was cued *immediately* at the offset of motion. As detailed in the Methods section, the observer adjusted the orientation of a pointer to report the perceived direction of motion of the cued target. While the observer had to hold in memory the direction of motion of this cued target during the adjustment phase, having a single item and no delay after stimulus offset minimized the involvement of memory capacity limits in the performance of the observer. This way, we sought to characterize the stimulus processing and encoding stages prior to memory storage.

**Figure 3:**
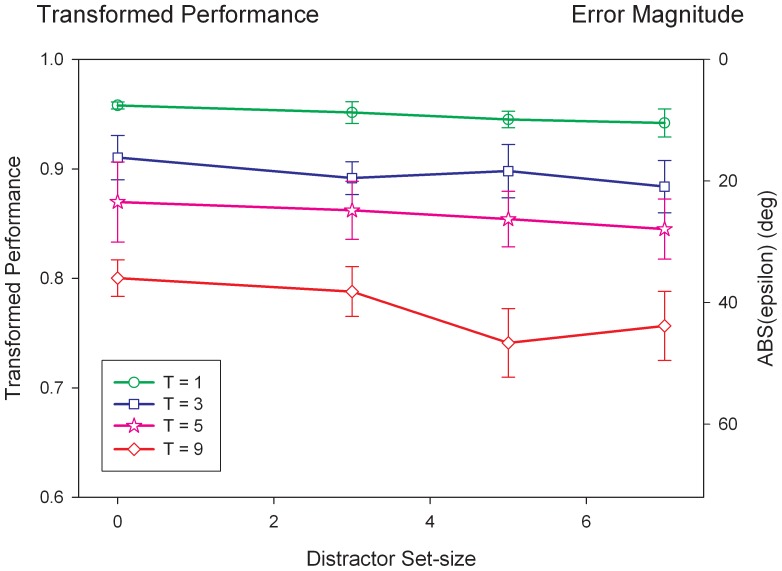
Stimulus encoding performance as a function of distractor and target set-sizes. The magnitude of the error angle 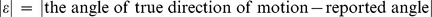 is shown on the right y-axis. The left y-axis shows the equivalent transformed measure defined as 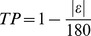. According to this transformed measure, 1 and 0.5 correspond to perfect and chance levels of performance, respectively. Although both target and distractor set sizes have a significant influence on performance, the effect of target set size is more pronounced. Data points correspond to the mean across observers (N = 4) and error bars represent ±1 SEM.



Fig. 3 plots performance for each target set-size as a function of distractor set-size. The magnitude of the error angle calculated as:
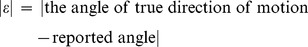
(1)is shown on the right y-axis. The left y-axis shows the equivalent transformed measure [43] defined as

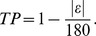
(2)According to this transformed measure, 1 and 0.5 correspond to perfect and chance levels of performance, respectively. A repeated measures ANOVA shows that target set-size (F(3,9) = 171.421, p<0.0001, η_p_^2^ = 0.950) and distractor set-size (F(3,9) = 16.576, p = 0.019, η_p_^2^ = 0.725) are significant but not their interaction (F(9,27) = 1.007, p = 0.459, η_p_^2^ = 0.251). To quantify the effects of targets and distractors, we fitted lines to data and obtained slopes (see Table 1). The slopes of transformed performance as a function of target set-size indicate a drop in performance between 2% and 2.5% per target item. By equations (1) and (2), these slopes correspond to an increase between 3.5 deg to 4.6 deg of error angle per target item. In comparison, the slopes of transformed performance as a function of distractor set-size indicate a much smaller effect: a drop in performance between 0.2% and 0.7% per distractor item, corresponding to an increase between 0.4 deg and 1.4 deg of error angle per distractor item.

**Table 1:** Results of linear fits to data from Experiment 1.

Performance vs. Distractor Set-size			
Linear Fit y = a*x+b			
	a	b	R^2^
T = 1	−0.00237	0.958	0.98944
T = 3	−0.00328	0.90823	0.76139
T = 5	−0.00352	0.87095	0.97978
T = 9	−0.00773	0.80043	0.71105
**Performance vs. Target Set-size**			
Linear Fit y = a*x+b			
	**a**	**b**	**R^2^**
D = 0	−0.01951	0.97235	0.99433
D = 3	−0.01973	0.96206	0.98381
D = 5	−0.02552	0.97441	0.99642
D = 9	−0.02274	0.95912	0.9953

Top: Transformed performance as a function of target set-size. Bottom: Transformed performance as a function of distractor set-size.

**Figure 4:**
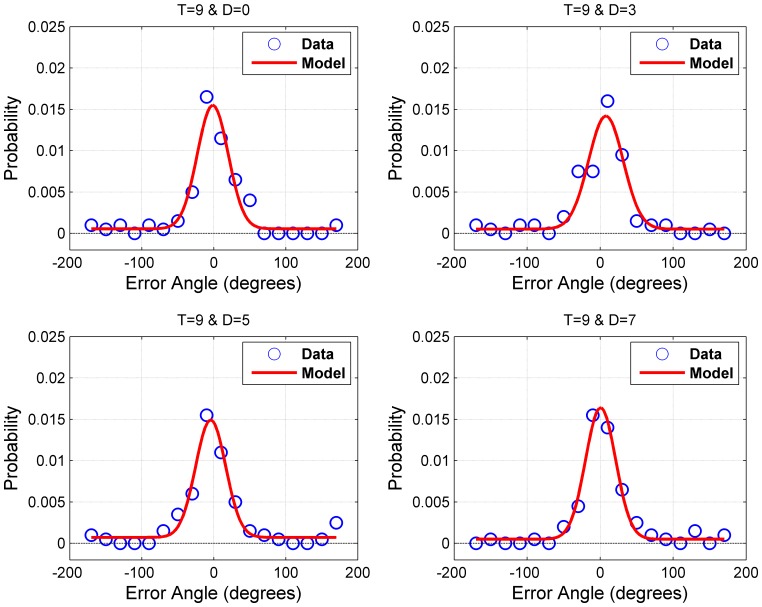
An example of the fit of the Gaussian+Uniform model to empirical error distributions for observer OEK at target set-size T = 9.

Thus, the drop of performance in this experiment reveals a clear bottleneck for target processing at the early stage of stimulus encoding. According to the leaky hourglass model, this bottleneck should be relatively minor compared to the bottleneck occurring in VSTM. To make this comparison, one can analyze this bottleneck in terms of its quantitative and qualitative limits [65]. Its quantitative limit, *intake*, refers to the fraction of target items that are processed. Typically the term “capacity” is used to denote the maximum number of items that can be processed or stored. Given the possibility that the number of items processed/stored can change depending on the precision of processing or storage, we use the term “intake” to describe the fraction of items processed or stored for a given stimulus. Its qualitative limit, *precision*, refers to the quality of encoding for the processed items. For example, a system may encode 10 items with low precision, or alternatively 4 items with high precision, depending on how resources are distributed or limited. In order to decompose performance into precision and intake measures, we fitted to our data a hierarchical family of descriptive statistical models. These models consisted of a Gaussian, Gaussian+Uniform, and Gaussian+Uniform+Misbinding models [12], [18]. We then compared different models in order to select the model with the best performance (details of these models and the selection process are available from the authors upon request). The selected model was a Gaussian+Uniform mixture model [18], defined as:
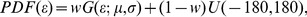
(3)where the probability density function *PDF(ε)* of errors (ε = the angle of true direction of motion – reported angle) is expressed as a mixture model of two distributions: 1) A Gaussian distribution *G(ε;μ,σ)* whose parameters represent the accuracy (mean: *μ*) and the precision (*1/σ*, where *σ* is the standard deviation) of encoding the direction of motion, and 2) a uniform distribution over the interval *(−180,180)* which represents guessing the direction of motion. The weight of the uniform distribution *(1-w)* represents the proportion of guesses across trials. The weight of the Gaussian, *w*, represents the proportion of responses to the target, which provides a relative measure for the intake of encoding. Figure 4 provides an example for the fits of the Gaussian+Uniform model to empirical data for one observer at target set-size of 9.

**Figure 5:**
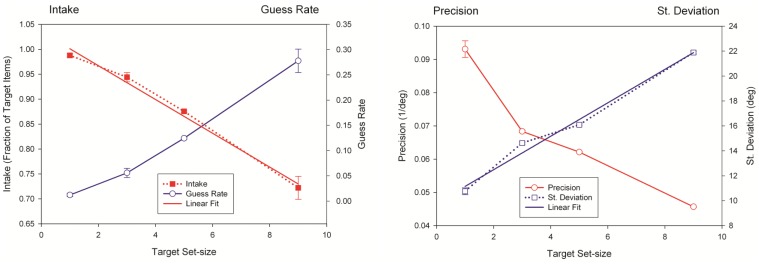
Precision (A) and intake (B) as a function of target set-size. Also included in the plots are guess rate (1-w) and standard deviation (σ). Note that the left and right y-axes have different offsets and scales. Data points correspond to the mean across observers (N = 4) and error bars represent ±1 SEM.

Figure 5 plots the precision (left axis shows the precision *1/σ* and the right axis shows the standard deviation *σ*) and intake (left axis shows intake *w* and the right axis shows the guess rate *1-w*) parameters of this model averaged across the observers as a function of target set-size. Linear relationships are observed between set-size and standard deviation as well as between set-size and the weight of the Gaussian.

In the next section, we carry out a similar analysis for memory stages and compare the bottlenecks occurring at different stages in order to test the leaky hourglass model.

### Experiment 2: Memory Stages

In order to compare the bottlenecks observed at the stimulus-encoding stage to those of subsequent memory stages, we repeated the previous experiment by inserting a delay between the end of the motion and the onset of the cue. Cue-delay values ranging from 0 s to 3 s were randomly interleaved from trial to trial. The case where cue-delay = 0 in Experiment 2 is similar to Experiment 1. However, Experiment 1 used exclusively cue-delay = 0 while 7 different cue delays ranging from 0 to 3 seconds were interleaved randomly from trial to trial in Experiment 2. The single cue-delay blocked-design of Experiment 1 allows observers to use a strategy optimized for this condition. Given that the stimulus duration was fixed, observers could predict when the cue would appear. In the randomly interleaved delay conditions of Experiment 2, subjects were required to spread their attention over time (since the cue delay in a given trial was not predictable) and possibly use a strategy where a non-selective transfer of information into memory occurs before a selective transfer [66]. Due to these differences, we used the blocked design approach for Experiment 1 for minimizing the involvement of memory processes.

**Figure 6:**
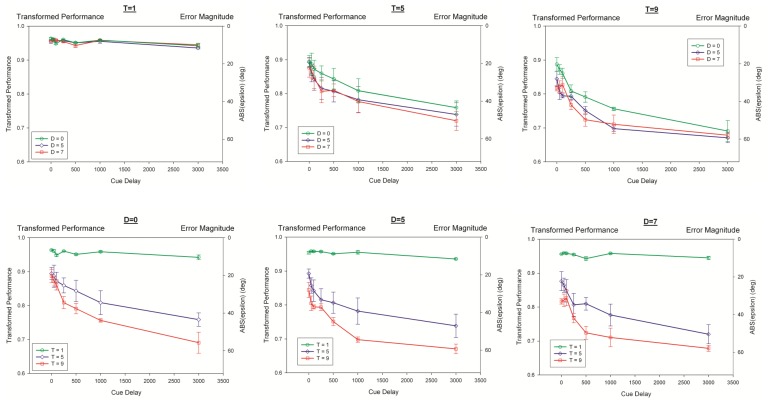
Transformed performance as a function of cue delay. In the upper row, each panel corresponds to a different target set-size. To show the difference between target and distractor effects, the lower row plots the same data with each panel corresponding to a different distractor set-size. Data points represent the mean across observers (N = 4) and ±1 SEM. Left and right y-axes are the same as in Fig. 3.

Figure 6 shows performance as a function of cue-delay. The effects of both target set-size (F(2,6) = 31.616, p = 0.002, η_p_^2^ = 0.913), distractor set-size (F(2,6) = 55.791, p<0.0001, η_p_^2^ = 0.949) and their interaction (F(4,12) = 5.272, p = 0.011, η_p_^2^ = 0.637) were significant.

**Figure 7:**
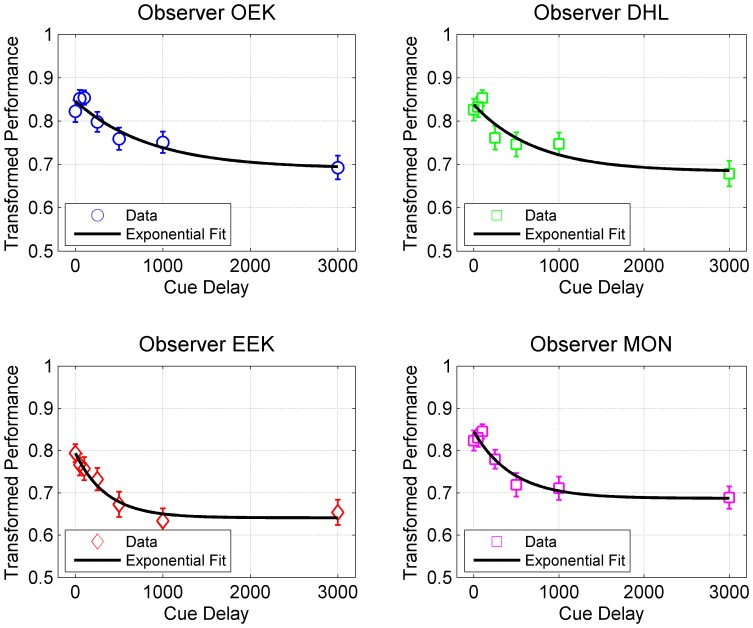
Transformed performance as a function of the cue delay for the condition in Experiment 2 with target set-size = 9 targets and distractor set-size = 7. Separate exponential fits are shown for the data of the 4 observers.

In agreement with previous studies of memory dynamics [1], [3], [41], [43], [66], [67], for set sizes >1 we find a rapid decay in performance. In the literature, the demarcation between sensory memory and VSTM is usually made by choosing a somewhat arbitrary delay value without taking into consideration stimulus parameters or individual subjects. However, this may not be accurate given that the dynamics of sensory memory depends on stimulus parameters and subjects (e.g., [2], [38], [39], [43], [68]–[71]). For demarcation, we applied the traditional definition of transient versus steady-state using the time-constants obtained from the fits to empirical decay functions. The observers’ transformed performance in Experiment 2 was fit by an exponential [38], [66], [68], [71] of the form
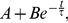
(4)where *t* is the cue delay, *A + B* represents transformed performance at *t*  =  0 *A* is the asymptotic performance as *t* approaches infinity, and 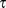 is the time-constant of the decay in performance. Figure 7 shows examples of exponential fits to the data and Table 2 provides the parameters for each subject for the case T = 9 and D = 7. By considering the case where the most pronounced drop in performance is observed (T = 9 and D = 7), the time-constants of the fits in Table 3 can be used to demarcate between sensory memory and VSTM, where VSTM represents the steady-state level of exponential decay. Using the traditional engineering definition of steady-state interval starting at the time when the response reaches ±5% of its asymptotic value, VSTM can be said to dominate at 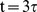. Accordingly, for the set of cue-delays used in our experiment, the cue-delay of 3 s corresponds to primary contributions of VSTM while the other cue delays correspond to primary contributions of sensory memory (except for observer EEK where a cue delay of 1 s is at the limit between sensory memory and VSTM). Note that, in doing this demarcation, we are not claiming that sensory memory and VSTM are purely sequential processes. The contents of sensory memory are read into VSTM *while* sensory memory is active. However, given the capacity differences between the two memory systems, at short (long) cue delays, average performance will be determined primarily by sensory memory (VSTM). Our samples of the cue delay consisted of 0, 50, 100, 250, 500, 1000, and 3000 ms. The goal of delineating sensory memory from VSTM is to decide which of the samples would involve primary contributions of sensory memory and which would be dominated by VSTM. While our analyses of sensory memory and VSTM depend on this demarcation, given that we have one sample at 1 s and another at 3 s, any shift in the demarcation within this interval does not affect our analyses.

**Table 2:** The results of the exponential fits to data from Experiment 2 for T = 9 and D = 7.

parameter	OEK	DHL	EEK	MON	All subjectscombined
A	0.69	0.68	0.64	0.68	0.67
B	0.15	0.15	0.15	0.15	0.15
τ (ms)	853.18	723.23	355.45	456.35	515.74
R^2^	0.90	0.85	0.96	0.92	0.95

**Table 3:** Results of significance tests and estimated effect size (η_p_^2^) for target and distractor set-sizes at each cue-delay in Experiment 2.

	Target Set-size (TSS)			Distractor Set-size (DSS)			Interaction TSS*DSS		
Cue Delay(ms)	F(2,6)	p	η_p_^2^	F(2,6)	p	η_p_^2^	F(4,12)	p	η_p_^2^
0	14.376	0.005	0.827	8.939	0.016	0.749	5.544	0.009	0.649
50	13.431	0.006	0.817	19.304	0.002	0.865	6.769	0.004	0.693
100	13.905	0.006	0.823	12.450	0.007	0.806	9.978	0.001	0.769
250	18.151	0.003	0.858	18.281	0.003	0.859	22.268	<0.0001	0.881
500	20.652	0.002	0.873	79.727	<0.0001	0.964	35.069	<0.0001	0.921
1000	32.589	0.001	0.916	17.180	0.003	0.851	1.903	0.175	0.388
3000	71.086	<0.0001	0.960	2.595	0.154	0.464	1.362	0.304	0.231

Given the significant interaction term between the effects of target and distractor set-sizes, in order to analyze if distractors interfere with memory during all of its temporal stages, we tested at each cue delay separately the effects of target and distractor set-sizes. Table 2 shows that target set-size is significant at all cue delays and the distractor set-size fails to reach significance only for cue delay of 3 s (i.e., only for VSTM). Taken together, these results show that distractor set-size was significant for each cue-delay within the duration of sensory memory but not for VSTM. Thus the filtering function of attention and its attendant limit play a major role only in the intermediate sensory memory stage where information is transferred and maintained from stimulus encoding to VSTM. This effect can be visualized in Fig. 6 by noting that data points for different distractor conditions converge to the (statistically) same point. This is not a signal-to-noise issue in terms of a floor effect, because performance at the convergence point is still higher than chance. A simple explanation is that distractors determine how fast information is transferred from sensory memory to VSTM and by the time 3 seconds have elapsed, there has been enough time for transfer, so that the speed of transfer does not matter anymore. The effect of distractors on the speed of information transfer can be understood within the framework of a selective transfer strategy. If the process consists of inspecting items to determine whether they are targets or distractors so as to transfer only targets into VSTM, then an increase in the number of distractors would imply an increase in the time required for inspection, thereby slowing down the transfer from sensory memory to VSTM.

**Figure 8:**
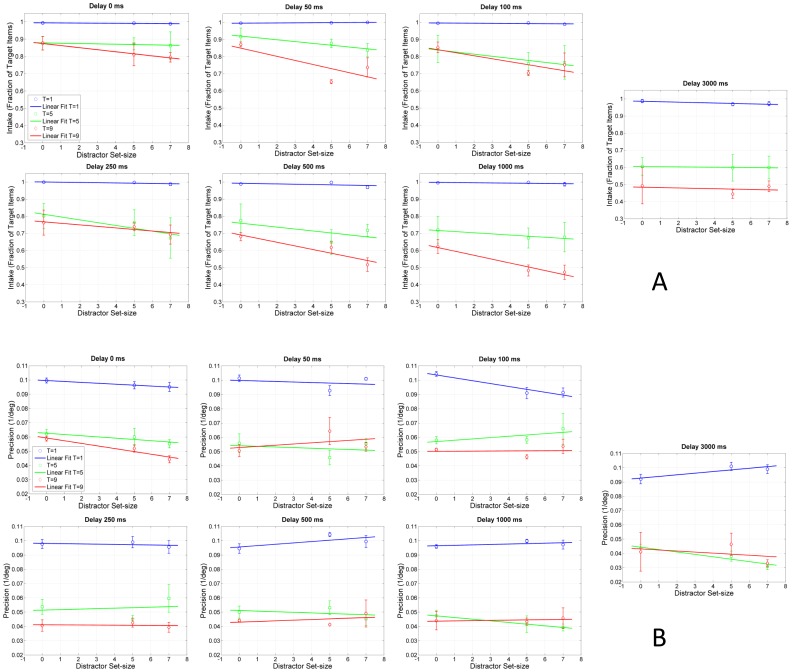
Precision (A) and intake (B) as a function of target and distractor set-sizes. Different panels represent different cue delays. Data points correspond to the mean across observers (N = 4) and ±1 SEM. Lines represent linear fits.

Sligte and colleagues suggested a modified memory model where a fragile intermediate form of VSTM takes place between large-capacity/high-resolution sensory memory and low-capacity/low-resolution VSTM ([72]–[74]; but see also [75], [76]). However, due the complex stimuli used in their change-detection paradigm, their decomposition of performance into quantity (capacity) and quality (resolution) was indirect [73]. A representation was called “high resolution” (cf. precision) when observers correctly detected a change in the display *and* correctly identified the changed object. In our study, we obtained more direct measures of memory intake and precision by using quantitative statistical models. The analysis of the data from Experiment 2 showed that the Gaussian+Uniform model was again the best performing model. Figure 8 plots the precision and intake parameters averaged across observers.

Increasing the target set-size causes precision to decrease, and unlike the stimulus encoding stage, saturation is observed for set sizes of 5 and 9. Precision does not depend on distractor set-size (F(2, 6) = 0.044, p = 0.957, η_p_^2^ = 0.015). Although increasing the target set-size causes a decrease in intake for all cue delays, distractors influence intake only for sensory memory. Taken together, our results agree with the finding that sensory memory requires attention [77] while showing, in addition, that the filtering function of attention is exclusive to the sensory memory stage in a specific manner, i.e., influencing only intake while sparing precision.

**Figure 9:**
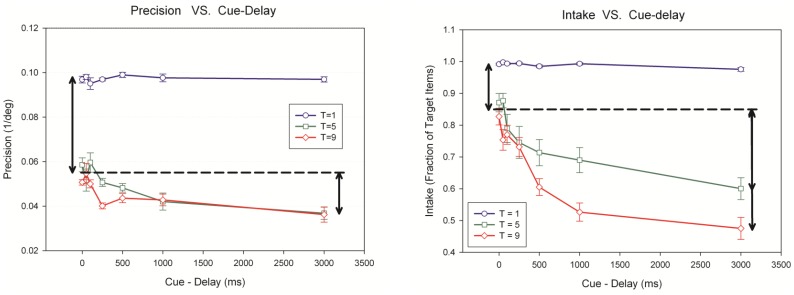
Precision (A) and intake (B) as a function of cue delay. The horizontal dashed line and the arrows in each panel highlight the relative share of drop in the quality (A) and quantity (B) of information between the stimulus encoding stage (cue delay = 0 s; leftmost data points) and VSTM (cue delay = 3 s, rightmost data points). Note that y-axes for the left and right panels start at 0.02 and 0.4, respectively. Data points correspond to the mean across observers (N = 4) and ±1 SEM.

Figure 9 plots precision and intake as a function of cue delay. The case of a single target provides a baseline (the best performance), which is largely independent of cue delay (for precision: F(6, 18) = 1.189, p = 0.363, η_p_^2^ = 0.284; for intake, there is a slight but significant change: F(6, 18) = 5.046, p = 0.003, η_p_^2^ = 0.627). As the number of targets is increased, one can observe the effect of bottlenecks. It is clear that the major bottleneck for the *quality* of information (precision) resides at the stimulus encoding stage rather than memory. This is highlighted in the left panel of Fig. 9 by the vertical arrow on the left positioned at cue-delay of 0 ms. Of the total precision drop of 0.06 deg^−1^, 63% to 75% (for T = 5 and T = 9, respectively) occurs at the stimulus encoding stage (cue delay = 0 s). The bottleneck for the *quantity* of information (intake) is more gradual, spreading with an exponential course from stimulus encoding to sensory memory and finally to VSTM. In this case, 32% of the total intake (for T = 5 and T = 9) drop occurs at the stimulus encoding stage.

## Conclusions

Capacity limits play a fundamental role in our conceptualization of cognitive function. Individual differences in capacity limits have been linked to individual differences in the performance of a variety of cognitive tasks [5]. The commonly accepted view is that the major bottleneck resides in VSTM as illustrated by the leaky hourglass analogy. As a result, most studies used a fixed cue delay designed to access VSTM and attributed the empirically observed bottlenecks to VSTM. In our study, as a first step, by cueing a *single* target item *immediately* at the offset of motion, we analyzed the capacity of stimulus processing and encoding stages *prior* to memory stages. Contrary to the predictions of the leaky hourglass model, our results show a significant quantitative and qualitative bottleneck at the stimulus encoding stage.

As mentioned in the previous section, Sligte’s and colleagues’ analysis of iconic memory and VSTM used an indirect way of quantifying capacity and precision [73]. In our study, we obtained more direct measures of memory capacity and precision by using quantitative statistical models. Another upshot of our study is that quality and quantity measures of information are subject to different bottleneck constraints. In terms of precision, the major bottleneck resides in stimulus encoding prior to memory stages. The bottleneck for intake is spread among the stimulus encoding and memorization stages.

**Figure 10:**
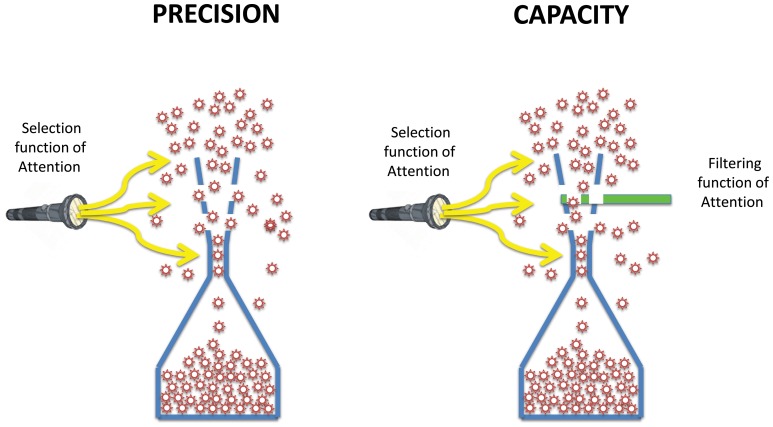
The Leaky Flask Model. The single leaky hourglass of Fig. 1 is replaced by two leaky flasks, one for precision and one for intake to highlight the different characteristics of these two aspects of bottlenecks. The top portions are narrower than the hourglass model to illustrate the bottlenecks occurring at the stages prior to VSTM. Also shown in this figure are the constraints imposed by attentional processes. While the selection function of attention applies to all three stages, the filtering function of attention applies mainly to the intake of sensory memory stage.

Similarly, the constraints introduced by attention should not be viewed as stemming from a unitary process. Our results show that while the selection constraint of attention applies to all three stages (stimulus encoding, sensory memory, and VSTM), the filtering constraint of attention applies mainly to the intake of sensory memory, sparing its precision. These findings are presented schematically by using a “leaky flask” model in Fig. 10.

Based on our findings, the single leaky hourglass is replaced by two leaky flasks, one for precision and one for intake to highlight the different characteristics of these two aspects of bottlenecks. The top portions are narrower than the hourglass model to illustrate the bottlenecks occurring at the stages prior to VSTM. Also shown in this figure are the constraints imposed by attentional processes. While the selection function of attention applies to all three stages, the filtering function of attention applies mainly to the intake of sensory memory stage. Taken together, our results provide a novel and detailed understanding of how multiple bottlenecks influence the processing of motion information during a single glance, from stimulus encoding to its transfer into sensory and short-term memory stores. The multiple bottlenecks seen in the current study are also likely to constrain performance in other tasks involving motion, such as traditional MOT [47].

## Supporting Information

File S1 **Modeling details.**
(DOCX)

File S2 **Bayesian statistics.**
(DOCX)
