# Supplementary material for: Bottlenecks of Motion Processing during a Visual Glance: The Leaky Flask Model
Source: PLoS One. 2013 Dec 31;8(12):e83671. doi: 10.1371/journal.pone.0083671 (PMC3877086; doi:10.1371/journal.pone.0083671)
Supplement: File S1 — Modeling details. (DOCX) [file pone.0083671.s001.docx]

`

SUPPORTING INFORMATION

S1. Modeling details

FOR

Bottlenecks of Motion Processing during a Visual Glance: The Leaky Flask Model

Haluk Öğmen^1,2*^, Onur Ekiz^1^, Duong Huynh^1^, Harold E. Bedell^2,3^, Srimant P. Tripathy ^4^

^1^Department of Electrical and Computer Engineering, University of Houston, Houston Texas, United States of America.

^2^Center for Neuro-Engineering and Cognitive Science, University of Houston, Houston Texas, United States of America.

^3^College of Optometry, University of Houston, Houston Texas, United States of America.

^4^School of Optometry and Vision Science, University of Bradford, Bradford, United Kingdom.

**S1. Modeling details**

To understand the performance of an observer in processing and/or memorizing information, one needs to quantify not only how many items are processed/memorized but also the quality associated with their processing/memorization. For example, a system can process 10 items with low precision, or alternatively 4 items with high precision, depending on how resources are distributed or limited. To introduce an assessment of both quality and quantity, we used the mixture model described in the text. In this model [1] subjects’ responses are treated as a function of a random variable. The distribution of errors is modeled by

$\mathrm{PDF}\left( \varepsilon\right)=w.G\left( \varepsilon;\mu,\sigma\right)+\left( 1-w \right)U\left( -180,180 \right)$ , (S1)


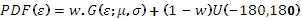


where the probability density function *PDF(ε)* of errors (ε=true direction of motion – reported direction of motion) is expressed as a mixture model of two distributions:

1. A *Gaussian* distribution *G(ε;μ,σ),* whose parameters represent the accuracy (mean: *μ*) and the precision (*1/σ*, where *σ* is the standard deviation) of encoding, and
2. a *Uniform* distribution $U$ over the interval *(-180,180),* which represents guessing.

This model provides an efficient decomposition of performance into qualitative and quantitative measures. The precision parameter *1/σ* captures the qualitative aspect of performance, with larger values of *σ* representing a larger spread of errors in the reported direction of motion. The weight of the uniform distribution *(1-w)* represents the proportion of trials on which the direction of motion was guessed rather than remembered. The weight of the Gaussian, *w*, represents the proportion of responses that is based on remembering some information of the target’s motion and this captures the quantitative aspect of performance. A nonlinear optimization routine was created using the Matlab fminsearch() function to estimate the parameters of this and the models described in the following section. Model outputs were calculated by using estimated parameters and compared to behavioral data. This nonlinear optimization routine was used to find the best fitting model for the distribution of behavioral data in each experimental condition.

**References**

1. Zhang W, Luck SJ (2008) Discrete fixed-resolution representations in visual working memory. Nature 453:233–235.
