# Supplementary material for: Bottlenecks of Motion Processing during a Visual Glance: The Leaky Flask Model
Source: PLoS One. 2013 Dec 31;8(12):e83671. doi: 10.1371/journal.pone.0083671 (PMC3877086; doi:10.1371/journal.pone.0083671)
Supplement: File S2 — Bayesian statistics. (DOCX) [file pone.0083671.s002.docx]

`

SUPPORTING INFORMATION

S2. Bayesian statistics

FOR

Bottlenecks of Motion Processing during a Visual Glance: The Leaky Flask Model

Haluk Öğmen^1,2*^, Onur Ekiz^1^, Duong Huynh^1^, Harold E. Bedell^2,3^, Srimant P. Tripathy ^4^

^1^Department of Electrical and Computer Engineering, University of Houston, Houston Texas, United States of America.

^2^Center for Neuro-Engineering and Cognitive Science, University of Houston, Houston Texas, United States of America.

^3^College of Optometry, University of Houston, Houston Texas, United States of America.

^4^School of Optometry and Vision Science, University of Bradford, Bradford, United Kingdom.

**S2. Bayesian statistics**


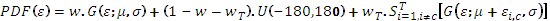


Here we provide the results of Bayesian analyses derived by using the repeated-measures ANOVA procedure described in Rouder et al. [1].

Table S1 shows Bayes factors for Experiment 1. The results are in agreement with ANOVA analysis; the highest Bayes factor is for the model where target and distractor set-sizes have independent effects. A model consisting of a target set-size effect only has also a very large Bayes factor, while the smallest Bayes factor belongs to a model containing only distractor set-size effect.

Table S2 gives the Bayes factors for Transformed Performance data in Experiment 2. The results are in agreement with the ANOVA analysis.

The results of the Bayes factor analysis for the same data but carried out individually at each cue delay are shown in Table S3. The results are in agreement with the ANOVA analysis with the exception of a cue delay of 100 ms, where the model with only a target set-size main effect had a higher Bayes factor compared to the model with both target and distractor set-size main effects. Nonetheless, the latter model had the second highest Bayes factor among all other models.

Table S4 shows the results of Bayesian analysis for the precision variable in Experiment 2. In agreement with ANOVA results, the model according to which precision depends on target but not on distractor set-size has the highest Bayes factor.

**References**

1. Rouder JN, Morey RD, Cowan N, Zwilling CE, Morey CC et al. (2008) An assessment of fixed-capacity models of visual working memory. Proc Natl Acad Sci U S A 105:5975-5979.

**TABLES**

| **Model** | **Bayes Factor** |
| --- | --- |
| T + S | 2.28917 e+21 ±0.82% |
| D + S | 0.1378796 ±0.67% |
| T + D + S | 9.612256 e+21 ±1.44% |
| T + D + T*D + S | 1.861683 e+21 ±1.14% |

**Table S1.** Bayes factors calculated following the procedures in Rouder et al. (2012) for the results of Experiment 1. T and D stand for Target and Distractor set-size fixed factors, and T*D represents their interaction. S stands for Subject as random factor. Bayes factors for these four models are calculated against the denominator consisting only of S.

| **Model** | **Bayes Factor** |
| --- | --- |
| T + S | 1.12319 e+53 ±1.03% |
| D + S | 0.294835 ±1.32% |
| CD + S | 110794.7 ±0.65% |
| T + D + S | 1.247476 e+54 ±2.13% |
| T + CD + S | 5.640296 e+78 ±1.14% |
| D + CD + S | 49430.65 ±7.87% |
| T + D + CD + S | 6.180784 e+81 ±2.88% |
| T + D + T*D + S | 2.894603 e+53 ±2.34% |
| T + CD + T*CD + S | 8.131872 e+90 ±5.64% |
| D + CD + D*CD + S | 287.2002 ±1.43% |
| T + D + CD + T*D + S | 1.079553 e+82 ±1.46% |
| T + D + CD + T*CD + S | 9.636877 e+95 ±1.91% |
| T + D + CD + D*CD + S | 6.368572 e+79 ±1.47% |
| T + D + CD + T*D + T*CD + S | 1.904058 e+97 ±2.73% |
| T + D + CD + T*D + D*CD + S | 1.248114 e+80 ±2.53% |
| T + D + CD + T*CD + D*CD + S | 1.527968 e+94 ±3.32% |
| T + D + CD + T*D + T*CD + D*CD + S | 3.167238 e+95 ±2.68% |
| T + D + CD + T*D + T*CD + D*CD + T*D*CD + S | 9.365581 e+93 ±2.47% |

**Table S2.** Bayes factors for the Transformed Performance data of Experiment 2. T, D, and CD stand for Target set-size, Distractor set-size, and Cue Delay fixed factors, and the terms with * represent interactions. S stands for Subject as random factor. Bayes factors for these models are calculated against the denominator consisting only of S.

| **Model** | **Bayes Factor** |
| --- | --- |
| **Cue Delay = 0 ms** | |
| T + S | 2292763 ±1.11% |
| D + S | 0.3860293 ±1.62% |
| T*D + S | 0.2570278 ±0.96% |
| T + D + S | 4275154 ±0.92% |
| T + T*D + S | 1209650 ±0.93% |
| D + T*D + S | 0.09689894 ±0.82% |
| T + D + T*D + S | 2900445 ±1.66% |
| **Cue Delay = 50 ms** | |
| T + S | 11382862 ±4.18% |
| D + S | 0.3306771 ±0.77% |
| T*D + S | 0.2322897 ±0.77% |
| T + D + S | 16536816 ±1.04% |
| T + T*D + S | 4495868 ±2.15% |
| D + T*D + S | 0.07999908 ±1.49% |
| T + D + T*D + S | 7682979 ±0.97% |
| **Cue Delay = 100 ms** | |
| T + S | 4220020 ±1.89% |
| D + S | 0.2908953 ±6.84% |
| T*D + S | 0.2433524 ±0.78% |
| T + D + S | 2767675 ±1.66% |
| T + T*D + S | 1975707 ±2.54% |
| D + T*D + S | 0.06862301 ±1.09% |
| T + D + T*D + S | 1429055 ±1.76% |
| **Cue Delay = 250 ms** | |
| T + S | 11004233261 ±1.01% |
| D + S | 0.2627469 ±0.92% |
| T*D + S | 0.2056753 ±0.75% |
| T + D + S | 14798858086 ±1.08% |
| T + T*D + S | 3444785649 ±1.34% |
| D + T*D + S | 0.05576218 ±1.43% |
| T + D + T*D + S | 4935594656 ±1.19% |
| **Cue Delay = 500 ms** | |
| T + S | 3.12866e+11 ±0.76% |
| D + S | 0.2632409 ±0.84% |
| T*D + S | 0.2116731 ±1.5% |
| T + D + S | 799425071162 ±1.9% |
| T + T*D + S | 125551111622 ±2.67% |
| D + T*D + S | 0.0551271 ±0.87% |
| T + D + T*D + S | 394935172019 ±5.9% |
| **Cue Delay = 1000 ms** | |
| T + S | 1.790571e+12 ±0.98% |
| D + S | 0.2265335 ±1.24% |
| T*D + S | 0.1994589 ±0.76% |
| T + D + S | 1.279347e+12 ±1.17% |
| T + T*D + S | 525886563317 ±1.23% |
| D + T*D + S | 0.04578123 ±3.21% |
| T + D + T*D + S | 393145455446 ±1.15% |
| **Cue Delay = 3000 ms** | |
| T + S | 1.963976e+16 ±1.12% |
| D + S | 0.1984695 ±1.71% |
| T*D + S | 0.1930719 ±0.79% |
| T + D + S | 6.700428e+15 ±0.99% |
| T + T*D + S | 5.214687e+15 ±0.87% |
| D + T*D + S | 0.0377539 ±1.61% |
| T + D + T*D + S | 1.867813e+15 ±2.41% |

**Table S3.** Bayes factors for the Transformed Performance data of Experiment 2 calculated separately at each cue delay. T and D stand for Target and Distractor set-size fixed factors, and T*D represents their interaction. S stands for Subject as random factor. Bayes factors for these models are calculated against the denominator consisting only of S.

| **Model** | **Bayes Factor** |
| --- | --- |
| T + S | 1.985338 e+93 ±1.17% |
| D + S | 0.04706041 ±1.85% |
| CD + S | 0.03811139 ±1.09% |
| T + D + S | 1.501016 e+92 ±2.37% |
| T + CD + S | 1.868724 e+97 ±0.98% |
| D + CD + S | 0.0017757 ±1.53% |
| T + D + CD + S | 1.585752 e+96 ±3.76% |
| T + D + T*D + S | 7.485058 e+90 ±1.94% |
| T + CD + T*CD + S | 1.208061 e+100 ±1.09% |
| D + CD + D*CD + S | 1.371086 e-05 ±3.7% |
| T + D + CD + T*D + S | 8.546515 e+94 ±1.82% |
| T + D + CD + T*CD + S | 1.113331 e+99 ±3.78% |
| T + D + CD + D*CD + S | 9.018579 e+94 ±1.34% |
| T + D + CD + T*D + T*CD + S | 6.946154 e+97 ±2.88% |
| T + D + CD + T*D + D*CD + S | 5.461086 e+93 ±3.07% |
| T + D + CD + T*CD + D*CD + S | 9.515606 e+97 ±2.97% |
| T + D + CD + T*D + T*CD + D*CD + S | 7.205084 e+96 ±18.89% |
| T + D + CD + T*D + T*CD + D*CD + T*D*CD + S | 5.070009 e+96 ±3.65% |

**Table S4.** Bayes factors for the Precision variable in Experiment 2. T, D, and CD stand for Target set-size, Distractor set-size, and Cue Delay fixed factors, and the terms with * represent interactions. S stands for Subject as random factor. Bayes factors for these models are calculated against the denominator consisting only of S.
